# Genomic consequences of a century of inbreeding and isolation in the Danish wild boar population

**DOI:** 10.1111/eva.13385

**Published:** 2022-05-17

**Authors:** Beril Yıldız, Hendrik‐Jan Megens, Christina Hvilsom, Mirte Bosse

**Affiliations:** ^1^ Animal Breeding and Genomics Wageningen University & Research Wageningen The Netherlands; ^2^ Department of Animal Ecology Netherlands Institute of Ecology (NIOO‐KNAW) Wageningen The Netherlands; ^3^ Copenhagen Zoo Frederiksberg Denmark; ^4^ Amsterdam Institute for Life and Environment (A‐LIFE) Section Ecology & Evolution Vrije Universiteit Amsterdam Amsterdam The Netherlands

**Keywords:** Danish wild boar, European wild boar, inbreeding, Klelund Plantation, ROH

## Abstract

Demographic events such as series of bottlenecks impact the genetic variation and adaptive potential of populations. European megafauna, such as wild boars (*Sus scrofa*), have experienced severe climatic and size fluctuations that have shaped their genetic variation. Habitat fragmentation and human‐mediated translocations have further contributed to the complex demographic history of European wild boar. Danish wild boars represent an extreme case of a small and isolated population founded by four wild boars from Germany. Here, we explore the genetic composition of the Danish wild boar population in Klelund. We genotyped all 21 Danish wild boars that were recently transferred from the source population in Lille Vildmose into the Klelund Plantation to establish a novel wild boar population. We compared the Danish wild boars with high‐density single‐nucleotide polymorphism genotypes from a comprehensive reference set of 1263 wild and domesticated pigs, including 11 individuals from Ulm, one of two presumed founder locations in Germany. Our findings support the European wild background of the Danish population, and no traces of gene flow with wild or domesticated pigs were found. The narrow genetic origin of the Danish wild boars is illustrated by extremely long and frequent runs of homozygous stretches in their genomes, indicative of recent inbreeding. This study provides the first insights into one of the most inbred wild boar populations globally established a century ago from a narrow base of only four founders.

## 1 INTRODUCTION

Population fitness can be negatively affected by inbreeding and other harmful genetic threats such as drift (Barrett & Charlesworth, 1991; Crnokrak & Roff, 1999; Hedrick & Kalinowski, 2000). Inbreeding increases the autozygosity throughout the genome due to consanguineous matings (Keller & Waller, 2002; Kim et al., 2015). A way to measure genomic autozygosity is by estimating runs of homozygosity (ROH) (Curik et al., 2014; Kim et al., 2015). ROHs are homozygous stretches along the genome caused by the transmission of haplotypes that are identical‐by‐descent (IBD) from parents to offspring (Keller et al., 2011). Longer ROHs reflect autozygosity due to recent common ancestors as shared IBD segments gradually break down overtime due to recombination (Bosse et al., 2012). Conversely, short ROHs usually represent autozygosity of ancient haplotypes that are fixed in the population (Ferenčaković et al., 2013; Kirin et al., 2010; Zhang et al., 2015). High levels of inbreeding often result in inbreeding depression when the rise of homozygosity and recessive harmful variants reduce the fitness of individuals (Bosse et al., 2019; Crnokrak & Roff, 1999; Zhang et al., 2015). Small and fragmented populations are especially prone to inbreeding (Lacy, 2000; Saccheri et al., 1998) and therefore at higher risk of reduced fitness due to inbreeding depression. In such populations, purging of detrimental alleles works less effective due to weaker selection and stronger drift (Keller & Waller, 2002; Pecnerova, 2018; Wang et al., 2021). Populations experiencing severe decline in numbers will experience fluctuations in the frequencies of alleles. Even deleterious recessive alleles may, as a result, fluctuate rapidly to high frequency (Robinson et al., 2018; Simons & Sella, 2016; Wang et al., 2021). Likewise, high drift influences the probability of adaptive alleles reaching fixation in small populations (Wilson et al., 2014). For instance, population bottlenecks increase the risk of adaptive mutations to become lost (Wahl et al., 2002; Wilson et al., 2014).

Population bottlenecks often result from founder events (Frankham et al., 1999; Simons & Sella, 2016). Founder effects can be particularly strong when a new population is established by only a few individuals (Parisod et al., 2005). Nevertheless, there are numerous examples of founder effects not leading to noticeable inbreeding depression. For instance, a small population of island foxes (*Urocyon littoralis*) recovered from founder bottlenecks through strong purging of deleterious alleles (Robinson et al., 2018). In another study, Xue et al. (2015) proposed that the endangered mountain gorillas are affected by recent autozygosity due to bottlenecks, noting that the population continued to persist through migration or effective purging of deleterious alleles by natural selection. This illustrates that low genetic diversity may not always result in an increased risk of extinction due to inbreeding depression. It is therefore important to investigate the genetic consequences of inbreeding in small and isolated populations to further predict their adaptability and persistence.

During the last glaciation, European wild boars suffered strong declines in genetic variation due to severe bottlenecks compared with more subtle effects on Asian wild boars (Groenen et al., 2012). More recently, wild boars in Europe substantially declined mainly due to habitat fragmentation and hunting pressures during the 1800s (Scandura et al., 2011). The wild boar was exterminated or highly marginalized in almost all European countries by the beginning of the 20th century (Apollonio et al., 1988; Celio Alves et al., 2010). Free‐ranging wild boars disappeared in Denmark in the beginning of the 19th century (Alban et al., 2005). However, one wild population was re‐established in the northern part of Denmark, in Lille Vildmose, Jutland. This population is known to be founded in 1926 by only two men and two women, from Ulm and Hagenbeck in Germany. Despite this extreme founder event, the population has persisted over the last century. From this population, a total of 21 individuals were translocated to Klelund Plantation in 2016 and 2017. No evidence exists for natural migration or deliberate stocking from elsewhere, although it cannot be ruled out either. Furthermore, it is unclear if all four founders have contributed to the population. Over the years, population size fluctuations resulted in a series of bottlenecks and expansions. Mean population size was estimated to be 181 individuals between the period 1988–2010. The population size has been more or less stable around 150 individuals since 2002 (Hald‐Mortensen et al., 2012).

The aim of this study is to unravel the genetic composition of the potentially highly inbred Danish wild boar population to gain insight into their demographic history. More specifically, we investigated the genetic background, potential hybridization with European domestic or wild boars, and the level of inbreeding in the animals that were the foundation of the Klelund population. Danish wild boars were expected to be genetically most similar to individuals from Ulm and Hagenbeck as the presumed founder populations. Gene flow from other wild boars was not expected as the Danish population is not in close geographic proximity to wild boar populations in Germany. The population was assumed to be wild, thus admixture with domesticated pigs was considered highly unlikely unless domestic pigs were released into the area or had escaped from nearby farms. However, earlier genetic introgression from domesticated pigs in the source population cannot be ruled out, since hybrids are widespread throughout North‐western Europe (Goedbloed et al., 2013). Considering its demographic history, we expected to find very high levels of inbreeding in the Danish population as there were only four founders that constitute the entire genetic basis of the population. Therefore, the Danish wild boars are likely to contain a high proportion of homozygous segments due to series of bottlenecks.

## 2 MATERIALS AND METHODS

### 2.1 Study population

Our study population is wild boars (*Sus scrofa*) from Klelund Plantation, Denmark. This population was established with the introduction of 12 individuals in 2016, with additional supplementation of nine individuals in 2017. All 21 individuals originated from the source population in Lille Vildmose, Jutland, Denmark, which was founded in 1926 by four founders, two of each sex. There is no information on the population size in Lille Vildmose at the time of the two translocations of 12 and nine individuals in 2016 and 2017, respectively. After the yearly spring population regulation in 2020, the source population size, that is Lille Vildmose, was assumed to be stable around 150 individuals, whereas the population size of boars in Klelund Plantation was around 65 pigs.

### 2.2 Samples and genotype data

We used PAX gene blood samples that were collected from 21 wild boars in Klelund Plantation. Additionally, 11 tissue biopsies were collected from wild boars in Ulm, Germany as a part of the yearly population cull by hunters. The SNP genotyping was conducted using the Illumina Porcine 60K+iSelect Beadchip, and a total of 61 565 loci were screened for each individual. We filtered out SNPs with overall call rate less than 95%. Due to missing genotype data, 1576 SNPs were removed. The final data set consisted of 48 227 SNPs. We did not remove SNPs based on minor allele frequency, even if MAF was zero, as we were interested to find the level of inbreeding of the Danish wild boars in comparison with other populations, and because of structure in our data set, this could lead to unequal removal of informative markers (Meyermans et al., 2020). On average, we found around 63 SNPs with a missing call rate, which corresponded to one in every ~755 SNP to be missing. The total length of the genome covered was 2436.3 Mb. The SNP density was found to be roughly one SNP per 50 kb. Total genome coverage for all individuals were higher than 98%, and the total genotyping rate was 0.998676. This information was further used for ROH settings.

### 2.3 Reference data set

To interpret our findings on population origin and inbreeding levels, we included other wild boar populations and domestic breeds across Eurasia. We obtained the reference samples and genotype data from (Iacolina et al., 2016, 2018; Yang et al., 2017a), which contains 1263 wild boars across Eurasia and international commercial pig populations (Table S4) (Ramos et al., 2009). We checked overlapping SNP markers, which corresponded to 47 325 variants in the combined data set. From the combined data set, a subset of only European wild boar populations consisting of 459 individuals was made to make more detailed comparisons. These data sets were used for population structure analyses (PCA and admixture).

### 2.4 Inbreeding levels and runs of homozygosity

We screened regions containing ROH using the homozygosity tool in PLINK 1.9 (Chang et al., 2015) to examine the level of inbreeding per individual (Kim et al., 2015). The size and number of ROHs was determined using a sliding window approach. ROHs were identified for all autosomes (18 chromosomes in total) of the 32 genotyped wild boars, representing 21 individuals from Klelund, Denmark and 11 from Ulm, Germany. The criteria to define ROHs using the –homozyg tool were as follows: (i) a sliding window of 20 SNPs (‐window‐snp 20); (ii) ROHs must contain at least one SNP per 100 kb on average (‐density 100); (iii) two consecutive SNPs must be less than 1000 kb apart (‐gap 1000); (iv) ROHs must be longer than 2000 kb (‐kb 2000); (v) one SNP allowed with missing genotype (‐window‐missing 1); and (vi) one possible heterozygous genotype (‐window‐het 1). No pruning was performed based on linkage disequilibrium (LD), but the minimum length of a ROH was set to 2 Mb to exclude short ROHs that derived from LD. Later, we confirmed the absence of ROHs shorter than 2 Mb in our study population by modifying the length of consecutive SNPs in homozygous segments. The choice of 20 SNPs per window roughly corresponded to one SNP per 100 kb. For our study, we allowed ROHs with one heterozygous SNP to account for any genotyping errors. We tested the impact of this criteria by performing another scenario where we excluded ROHs with any heterozygous SNP, which only excluded 31 ROHs.

To display ROH segments, we selected three chromosomes that were most informative. Chromosome 1 is the largest chromosome found in wild boar and other species (Bosse et al., 2014), chromosome 7 showed higher variability than average across all chromosomes and contains important immune gene families, and chromosome 15 consisted of very long ROHs (>50 Mb), the shortest being ~4 Mb. In addition, population inbreeding levels were computed using the genomic inbreeding coefficient F_ROH_ (Ferenčaković et al., 2013), defined as the total ROH length divided by the length of genome covered by SNPs (Kim et al., 2015; Mastrangelo et al., 2017). A non‐parametric test, Mann–Whitney U, was used to test F_ROH_ and total ROH size differences in wild boars from the Klelund Plantation and Ulm. We used an independent samples t‐test to compare average ROH sizes in both populations. All statistical analyses and graphs were performed R version 4.0.4 (R Core Team, 2021).

### 2.5 Population structure

We inferred proportions of shared ancestries in three approaches. First, to detect underlying genetic structure, we performed a Principal Component Analysis (PCA) in PLINK 1.9 (Chang et al., 2015) based on the allele frequencies between individuals from various populations (Ma & Amos, 2012). As a complementary analysis, we computed pairwise identity‐by‐state distance using the SNPRelate R package (version 1.24.0) (Zheng et al., 2012). Then, hierarchical clustering was conducted on 194 Western European wild boar samples using 47,871 SNPs. Groups were determined by permutation with a Z‐score threshold of 15 and outlier threshold of 5. This created 13 groups. Admixture analysis was also performed to assess population structure the most likely number of genetic clusters using the maximum likelihood approach (Muzzio et al., 2018; Skotte et al., 2013) given *K* ancestral populations ranging from 1 to 8 with cross‐validation errors using the package ADMIXTURE 1.3.0. (Alexander & Lange, 2011). The results were visualized as a bar plot of ancestry compositions in Rstudio (Leppälä et al., 2017; Lindqvist & Rajora, 2019).

## 3 RESULTS

### 3.1 Inbreeding levels and runs of homozygosity

We found 44% of 49,646 SNPs to have minor allele frequency of zero in the study populations. Average number of ROHs were much higher in the Klelund Plantation, Denmark compared to Ulm, Germany (Table 1). The average ROH size (*t*‐test, *p* < 0.05) and total ROH length (Wilcoxon test, *p* < 0.001) were significantly different between the two populations. Moreover, the genomic autozygosity (F_ROH_) in the Ulm population was consistently lower compared with Danish wild boars (Table S2). We found significantly higher percentages of total genome covered by ROHs (Wilcoxon test, *p* < 0.001) in Klelund Plantation, Denmark compared to Ulm, Germany (Table 1).

**TABLE 1:** ROH characteristics for the study populations, including average number of ROHs, average ROH length (Mb), average of the total ROH length (Mb) over individuals, ROH length span (Mb), and average genomic inbreeding coefficient (F_ROH_)

Population	Average no. of ROHs	Average ROH length (Mb)	total ROH length (Mb)	ROH length span (Mb)	Average F_ROH_
Klelund Plantation, Denmark (*N* = 21)	89.1	15.3	1344	2.8–155.9	0.568
Ulm, Germany (*N* = 11)	20.3	12.8	275	3–142.8	0.117

Note

F_ROH_ is an estimate of the overall genomic autozygosity disregarding runs shorter than 2 Mb.

**FIGURE 1:**
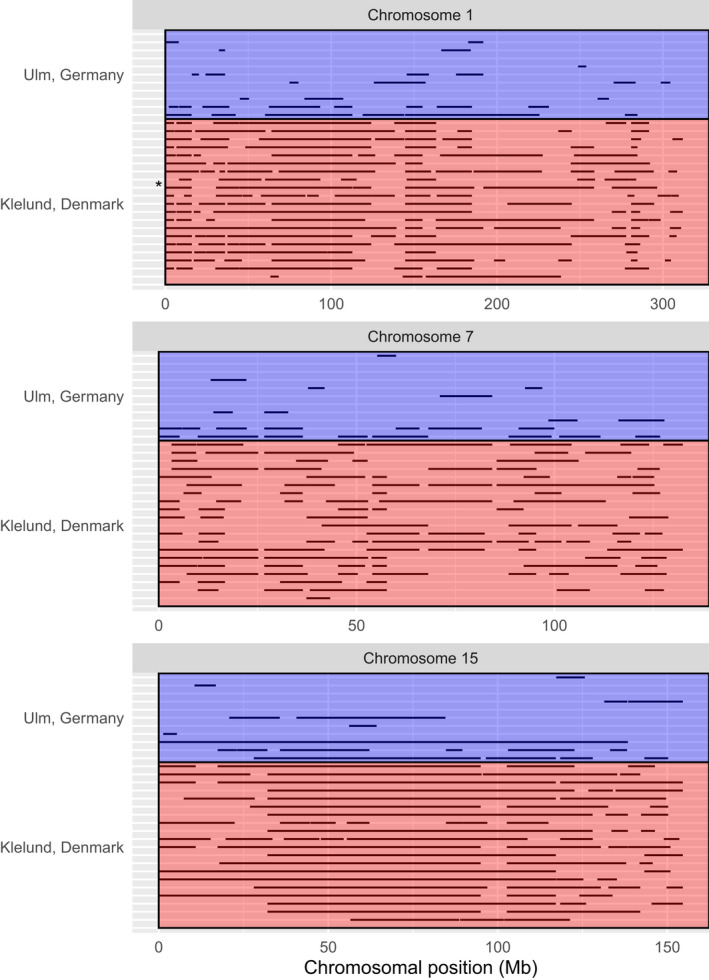
Chromosomal distribution of the ROH segments of the study population consisting of wild boars from Ulm, Germany and Klelund Plantation, Denmark. The x‐axis is scaled according to the length (in Mb) of each chromosome. ROH segments were presented on chromosome 1, chromosome 7, and chromosome 15. The length of each chromosome is 314.9 Mb, 134.7 Mb and 157.4 Mb for 1, 7 and 15, respectively. Individuals from the Danish population are highlighted as red, whereas individuals from Ulm in Germany are highlighted as blue. One wild boar from Klelund, Denmark with ROH segments covering 80% of chromosome 1 is shown with an asterisk

The extent of inbreeding was plotted as stretches of ROHs along all chromosomes for all individuals (Figure S1). The presence of stretches of ROHs per individual for chromosomes 1, 7, and 15 are illustrated in Figure 1. On chromosome 1, a Danish individual (shown with an asterisk) was found to be 80% homozygous with a total ROH length of 253 Mb on this chromosome (Figure 1). Moreover, another Danish individual contained the longest consecutive ROH segment (155.7 Mb) found in any of the populations across all chromosomes. On chromosome 7, ROH coverage of the Danish individuals (59.6%) was much higher than in individuals from Ulm (13.8%). ROH segments appear shorter and scarcer on chromosome 7, indicating a more heterozygous chromosome with ROH coverage of 46.1% for Danish and 9.2% for Ulm wild boars (Figure 1). Lastly, chromosome 15 was the least heterozygous chromosome with ROH segments encompassing 74.5% and 24.9% in the Danish and Ulm populations, respectively (Figure 1). In addition, the longest ROH segment (138.4 Mb) on chromosome 15 was found in a wild boar from Ulm. This individual had only one stretch of ROH and was almost entirely homozygous (87.9%) for chromosome 15. Similarly, almost half (42.7%) of the ROH segments across individuals on chromosome 15 were longer than 20 Mb.

**FIGURE 2:**
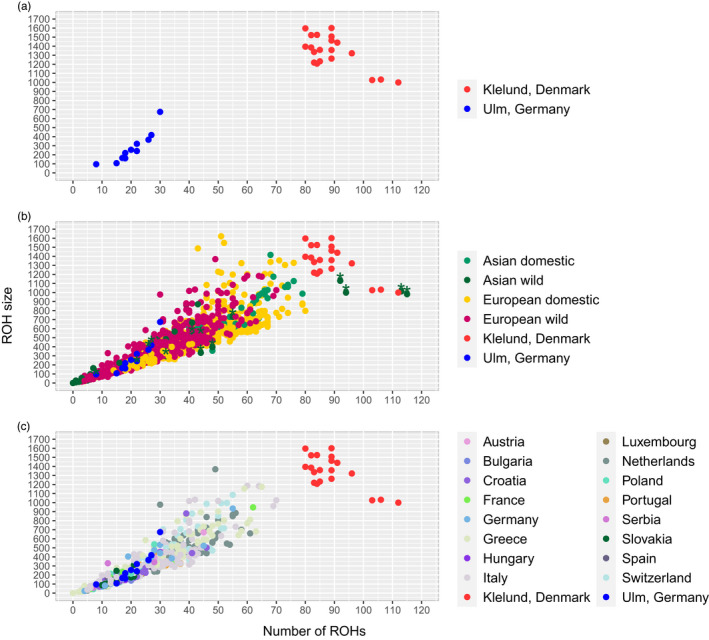
The relationship between the number of ROHs vs. total ROH size (Mb) in (a) the study populations consisting of wild boar populations from Klelund Plantation, Denmark and Ulm, Germany (b) comparison between global pig dataset with 1263 individuals and the study populations. Japanese wild boars are shown with an asterisk, and (c) comparison between the study populations and 459 individuals from other European wild boar populations

**FIGURE 3:**
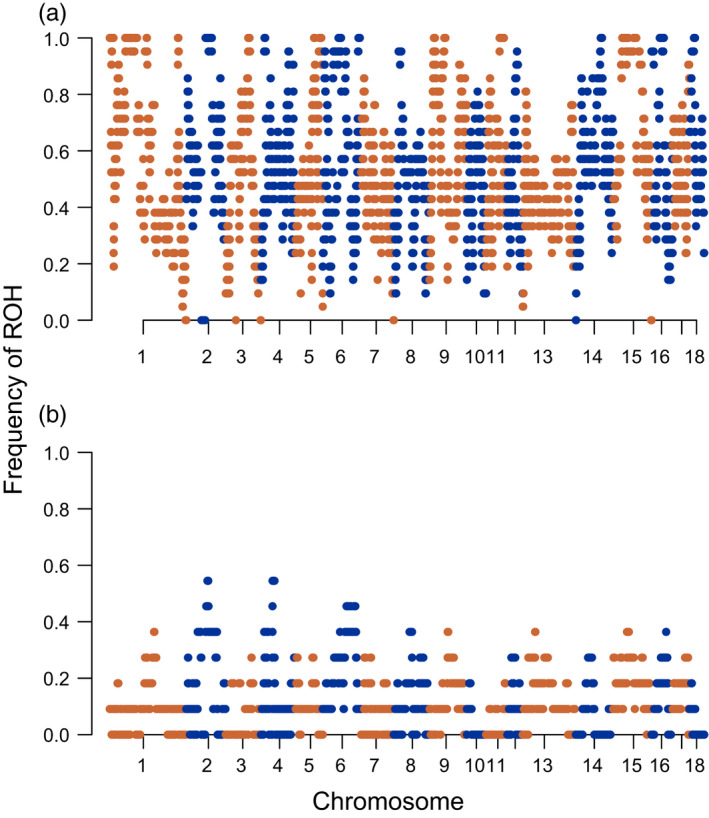
Manhattan plot of the frequency of ROH hotspots across the genome in wild boars from (a) Klelund Plantation, Denmark (*N* = 21) and Ulm, Germany (*N* = 11). The x‐axis represents the SNP positions along the chromosomes, and y‐axis shows the frequency (%) of overlapping ROH shared among individuals

We compared the frequency and size of ROHs in our study populations to those of the global reference data set consisting of wild boar populations and domestic breeds across Eurasia, and to a subset consisting of only European wild boars (Figure 2). The sum of ROHs in the Klelund population ranged from 1.05 Gb to 1.63 Gb, whereas in the Ulm population, the range was between 111.6 Mb and 674.4 Mb (Figure 2a). We found the highest number of ROHs in the Klelund and in some Japanese wild boar populations (Figure 2b, shown with asterisk). Although two individuals from the Japanese wild boar population had the longest total ROH size (1012 Mb and 1129.5 Mb). However, all wild boars from the Klelund population had 80 or more ROHs with a total length of over 999 Mb. Therefore, Danish wild boars have strikingly longer total ROH when compared with all groups, revealing the most extreme ROH coverage of any wild boar population studied to date. Other populations with long and frequent ROHs were from numerous European domestic breeds (e.g. Mangalica and Gloucester Old Spot), which had combined ROHs larger than 800 Mb similar to the Asian domestic breed, the Chinese Meishan pig (>500 Mb). Conversely, wild boar populations with shorter ROHs (<20 Mb cumulative) were from Russia, Greece and China. Furthermore, ROH comparison with the European wild boar data set (Figure 2c) revealed the Danish population as a distinct group in both sum and number of ROHs. Wild boars from Ulm were comparable to other European wild boars in both data sets, except one highly inbred individual. In the European wild boar data set, the longest cumulative size was found in a wild boar from Veluwe (1368.4 Mb), the Netherlands. Moreover, the highest number (70 runs) was identified in a wild boar from Sardinia, Italy with a cumulative ROH size of 1026 Mb. All members of the Danish population showed more extreme values in both number and total size of runs. We visualized the ROH frequency across total ROH length (Mb) categories in Figure S3, where the high contribution of Danish wild boars to large size categories is evident. We further explored the frequency of a SNP within ROH across the genome for our study populations (Figure 3). Danish wild boars showed numerous ROH islands containing all individuals (*N* = 21). Chromosome 15 showed almost half of the SNPs (1050 out of 2581) to have a frequency above 90%, while the majority of the SNPs (1782 out of 2965) on chromosome 7 had a frequency below 50% (Figure 3a). We found the highest frequency of ROH (54.5%) present in six individuals from the Ulm population (N=11) on chromosome 2 (Figure 3b). In addition, we found over 1/3 of the SNPs (15 753/47 072) with no ROH in individuals from Ulm, Germany. Strikingly, only 190 SNPs of 46,540 were not found within a ROH in Danish wild boars.

### 3.2 Population structure

**FIGURE 4:**
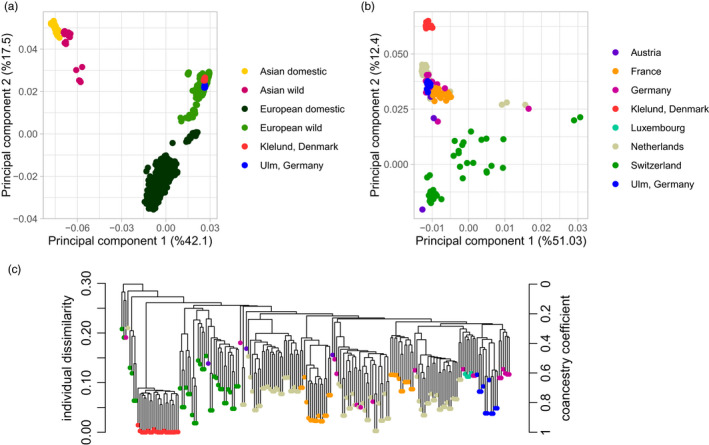
Principal component analysis (PCA) using 43 121 SNPs. (a) Four main groups consisting of 1263 individuals were established from the global reference data, namely; Asian domestic, Asian wild, European domestic, and European wild. The fraction of the total variance explained was 42.1% for eigenvector 1 (PC1) and 17.5% for eigenvector 2 (PC2). (b) 194 wild boar individuals from Western European populations. (c) Hierarchical clustering dendrogram based on pairwise identity‐by‐state (IBS) values of the Western European wild boar populations. Branch height represents dissimilarity on the left and co‐ancestry coefficient on the right

Population structure revealed by PCA based on SNP data clearly showed four distinct clusters (Figure 4a). The geographic origin of the global data correlated well to the observed SNP variation. The Danish and Ulm populations clustered closely to the other European wild boars in the PCA and formed the European wild boar cluster (Figure 4a). Furthermore, the first principal component largely mirrored the geographical continental distribution of the European and Asian groups, while the second component roughly showed the differentiation of the wild and domestic breeds within each geographic group (Figure 4a). The PCA including only the Western European wild boar populations showed a more detailed structure of the study populations (Figure 4b). Here, we see a clear separation of individuals from Klelund population from the rest, which is a result of severe inbreeding. In addition, it is known that European wild boar population has been subject to multiple restocking events that resulted in mixing gene pools (Scandura et al., 2008). We observed that wild boars from Switzerland scattered the most on both PCs as this is a very heterogeneous population with influences from North and South of the Alps. The most distinguishable cluster after Klelund displayed most countries including individuals from Ulm, Germany within the same group as those from Germany, the Netherlands, and France. Similar results were obtained when performing PCA including all European wild boar populations (Figure S2). The hierarchical clustering dendrogram of the Western European populations reflected the same clustering patterns as the PCA. Consistent with other plots showing genetic relationships, the Ulm population showed high similarity to other German wild boars while Klelund population did not co‐cluster with any populations and formed an outgroup. The scattering on PC1 reflects hybrid individuals that are mostly are from Switzerland.

**FIGURE 5:**
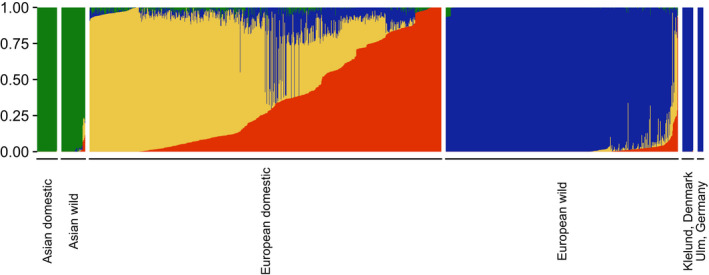
Admixture plot of 1263 individuals from the global pig data and the study population. Ancestral populations, namely K = 4 is chosen as the most biologically relevant. Four main groups were used from the Global pig data, namely; European Wild, European Domestic, Asian Wild, and Asian Domestic

**FIGURE 6:**
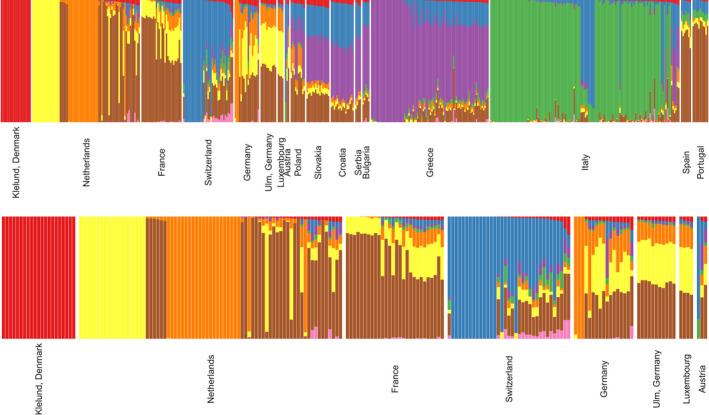
Admixture plot of K = 8 (a) 459 individuals from European wild boar populations. (b) 194 individuals from only Western European wild boar populations. The full plot of K = 2 to K = 8 of the European wild boars and cross‐validation error are shown in Figures S4 and S5

The admixture plot matched well with the PCA clusters, with distinctions between geographical origin and domestication status within Europe with K = 4 (Figure 5). The pairwise F_ST_ estimates between clusters reflected the degree of divergence between clusters (Table S1). Clusters 3 and 4 showed the lowest divergence (0.114). Wild boars from Ulm and Denmark only contained one ancestry component in line with their presumed European wild background. Overall, we found no traces of admixture with pigs. In the detailed European data set, wild boars clustered according to country of origin and geography (Figure 6). Again, a clear separation of the Klelund population is observed with one gene pool as a consequence of being a highly inbred and homogenous population, with no signs of admixture. The Ulm population was in close genetic proximity to the German wild boars and individuals from the Netherlands, also revealing more substructure.

## 4 DISCUSSION

We provide the first genome‐wide survey of a Danish wild boar population originating from a source population founded by four individuals in 1926 and subsequently experienced population fluctuations, shaping their genetic diversity. Our results showed extremely high inbreeding as a consequence of demographic events such as the founder effect in a small and isolated population. It is known that European wild boars contain overall lower genetic variation (Bosse et al., 2012) and higher mutational load (Bosse et al., 2019) than Asian wild boars and domestic breeds. It has been suggested, that the historically lower variation in European wild boars is due to the more notable population size decline during the last glacial period (Alexandri et al., 2017; Groenen et al., 2012; Iacolina et al., 2016). More recently, increased hunting pressures and restocking activities following demographic declines further shaped the genetic structure of the European wild boar (Scandura et al., 2008; Vilaça et al., 2014). These restocking events that affected local gene pools were largely uncontrolled and did not always result in favourable conditions (Frantz et al., 2013; Vernesi et al., 2003).

We investigated inbreeding levels in wild boars from the Klelund Plantation using ROH size as a predictor for the time of inbreeding (Ferenčaković et al., 2013; Wang et al., 2021), and the genome‐wide ROH coverage as an indication of inbreeding levels. Prior to ROH analysis, we identified almost half of the SNPs not being variable. Although, the SNP chip is designed for pigs and therefore would result in some loss of variation, it also indicates the presence of large homozygous regions, unless the study population contained pig hybridization.

The Danish population in the Klelund Plantation illustrates a unique case of extreme inbreeding, caused by an exceptional bottleneck followed by many generations with a small population size and without any apparent opportunity for gene flow from other wild boar populations. The Klelund population therefore provides a unique opportunity to study the genomes of a population of large mammals for the consequences of extreme demographics, such as the accumulation of long ROH segments. ROHs longer than 8 Mb are presumably due to autozygosity of recent origin, where there would be limited opportunity for recombination to break up the homozygous segments (Ferenčaković et al., 2013; Kirin et al., 2010; Purfield et al., 2017). This is also in congruent with relatively limited recombination events given that the population was established less than hundred years ago.

We found individuals with exceptionally high levels of homozygosity, in many cases covering almost the entire chromosome length, as visible in chromosome 1 and 15. Interestingly, chromosome 7 has been shown to contain fewer and shorter ROHs compared with other chromosomes. In pigs, chromosome 7 contains the Major Histocompatibility Complex (MHC) and other gene families that have important functions in immunity (Dadi et al., 2015; Schwartz et al., 2018). The MHC in vertebrates has often been shown to confer heterozygote advantage (Aguilar et al., 2004; Oliver et al., 2009). Preserved MHC diversity, as a consequence of selection from pathogens, may have been one of the underlying mechanisms that helped the Danish wild boar to persist through generations. The MHC has also been implicated to be involved in active inbreeding avoidance, and therefore even in the absence of rotating pathogen burden, may lead to active maintenance of diversity at this locus (Santos et al., 2016). Our study clearly suggests that an active mechanism of preservation of MHC locus diversity is at play, and a more detailed analysis of those mechanisms is warranted.

Overall, our findings suggest that Danish wild boars in the Klelund Plantation are extremely homozygous, while other European wild boars showed shorter and less frequent ROHs. The shorter ROH size found in the extensive European wild boar data set has been proposed to be linked to reduced historical population size and subsequent bottleneck effects due to climatic fluctuations occurring since the last ice age (Groenen et al., 2012), followed by bottlenecks and low levels of inbreeding in the last century (Apollonio et al., 1988; Herrero‐Medrano et al., 2013). These bottlenecks may have resulted in reduced N_e_ in European wild boars. A study by Yang et al. (2017b) revealed N_e_ between 44 and 67 for the European wild boar populations, with North‐Western European wild boar being the lowest. So far, the most severe case of inbreeding was detected in Japanese wild boars inhabiting very small islands (Bosse et al., 2012). Such small and fragmented populations are likely to suffer from very low levels of within‐population diversity due to cumulative effects of increased drift and reduced fitness. This illustrates the importance of local population size as it may overrule geographical location and relatedness. It is also important to note that ascertainment bias could influence the diversity measures as the SNP chip is primarily designed for European domestic pigs (Ramos et al., 2009). All considered, ROH analysis using whole genome sequencing of wild boars from the Klelund Plantation would help to provide estimates of genome‐wide inbreeding levels in the population.

The population structure analyses positioned Danish wild boars from the Klelund Plantation in the European wild boar cluster. In line with Novembre et al. (2008), PCA results hinted a correlation between the genetic and geographic distance between populations. In the PCA including wild boars from Western Europe only, the Klelund population constitutes a distinct group separate from the other Western European populations. This was expected, as we know it is an isolated population that experienced size fluctuations. Surprisingly, the proposed founder population from Ulm, Germany clustered with other geographically nearby populations and formed another distinct cluster. Therefore, it seems likely that there has been gene flow between these neighbouring populations leading to high levels of admixture among them, but with no indication of ancestry sharing between the Ulm and Klelund populations.

The admixture results from the global pig data suggested that there was no gene flow from domestic pigs into the study population. Note that this conclusion may largely be influenced by recent genetic effects, such as strong drift or inbreeding and therefore potentially distort historical relationships by presenting such populations to have a more distinct composition (Lawson et al., 2018). The narrow genetic basis and high homogeneity of the Danish wild boars, in combination with the absence of any identification of admixture with other European populations, indicates no reintroduction or recent migration events after the population was established in 1926. Moreover, European domestic breeds had overall more substructure than wild boars, reflecting their complex and independent domestication history, different breeding histories and admixed nature (Yang et al., 2017a). The admixture plot with the Western European populations also did not reveal any gene flow from other populations, corroborating that wild boars in Klelund originate from one founder event.

Despite high genetic homogenization, the managers considered the Klelund population to have good health condition. The wild boars in the Klelund Plantation have an overall good population dynamic with a stable growth rate (personal communication Klelund Plantation manager). In European wild boar populations, the mean number of piglets and the survival % of piglets ranges from 3.5–6.9 to ~65%–93.7%, (see Table S3). There are no anomalies observed regarding litter size and piglet survival in the Klelund population, and no diseases, malformations or signs of inbreeding depression have been detected. Number of piglets per sow is even higher than in Southern European countries, which is in line with a North‐South cline (see Table S3). However, the piglet survival in Tofte seems low. Note that these numbers could be masked by supplementary feeding (Malmsten et al., 2017), age of first reproduction (Gethöffer et al., 2007; Orłowska et al., 2013), and potential hybridization with domestic pigs (Frauendorf et al., 2016). Therefore, we speculate whether the Danish wild boar population supports the hypothesis that not all small and isolated populations suffer from inbreeding depression and conclude that more research is needed to better understand the complexity of population dynamics and what makes them healthy. Here, we illustrate that despite an extreme founder effect, the population appears viable. There is growing evidence that after an initial bottleneck, small populations often tend to purge some of the deleterious variants (Goodnight, 1988; Grossen et al., 2020; Mathur et al., 2021). Various studies (Kyriazis et al., 2019; Robinson et al., 2018; van der Valk et al., 2019; Xue et al., 2015) have proposed the role of purging of highly deleterious variants in small and isolated populations as an underlying mechanisms for their persistence with low levels of genetic diversity. Island foxes provides a similar case to Danish wild boars in terms of low levels of heterozygosity and small population size resulted from isolation and strong bottlenecks, for which Robinson et al. (2018) suggested highly effective purging of harmful recessive mutations. This could be the scenario after the initial bottleneck experienced by the population. Recent bottlenecks have been shown to result in higher frequencies of deleterious mutations relative to populations that have remained small over longer time scales (Bortoluzzi et al., 2020). Past bottlenecks during the last ice age have been shown to result in increased frequency of deleterious variants outside ROH regions in the European wild boar (Bosse et al., 2019). Similar to the Alpine ibex, Danish wild boars might have been successful in purging those mutations that are highly deleterious as a result of bottlenecks (Grossen et al., 2020; de Jong, 2018). Therefore, these bottlenecks may have contributed to purging of load, which would increase the chances of some founders being successful in establishing a novel population with small N_e_. Population demographic history of wild boars show high efficiency in range expansions associated with post glacial recolonization (Liu et al., 2019), which may suggest that Danish wild boars are not an exception when compared to other wild boar populations. The potential to expand after the founder event and a stable number of animals after expansion may have facilitated purging. Examining the efficiency of purging of deleterious mutations has large potential in providing explanations for preventing inbreeding depression and the persistence of small populations. Sequencing the whole genome would help to disentangle the genetic load and in examining potentially harmful mutations that have stayed in the population over 20 generations since 1926. A recent study on a small and highly inbred grey wolves population on Isle Royale investigated the genetic basis of inbreeding depression and found large ROHs associated with higher homozygosity of deleterious variants (Robinson et al., 2019). These extreme ROH lengths, with homozygous deleterious alleles, resulting from recent common ancestors may have detrimental effects on population viability in the long term. Further investigations on the phenotypic consequences of such high levels of inbreeding would be beneficial for the management of the Klelund Plantation. Predicting future threats, such as inbreeding depression, could be achieved via population genetic simulations based on past and current demographics and through investigating levels of deleterious mutations and genetic variation across generations (Boakes & Wang, 2005; Kyriazis et al., 2020).

Demographic histories shape the genetic structure of differentiation between populations (Auton et al., 2009; Liu et al., 2019; Xue et al., 2015). Management strategies for extant populations should build from knowledge on past events, where factors affecting genetic diversity including inbreeding levels, effective population size and gene flow are regularly monitored. Reintroduction projects are a powerful way to restore genetic diversity within wild populations that are fragmented. For future reintroductions, the use of genomics would be beneficial to identify the best suitable donor population by investigating genomic compatibility, relatedness to the Danish population, and the level of inbreeding in the potential donor population (Frankham, 2010; Latch, 2020). The scimitar‐horned oryx (*Oryx dammah*), which was previously declared extinct in the wild, is an example of a successful use of genomics in conservation management to generate a large‐scale reintroduction project by a careful selection of founders from captive populations (Humble et al., 2020). Similarly, by thorough planning fragmented wild boar populations can undergo population restoration. There is growing potential for expanding the range of wild boars. Management towards improving connectivity between areas and increasing suitable habitat could initiate the use of local populations as potential founders that will propel their traits into new areas when conditions for range expansion are good.

This study provides the first insights into the demographic history and genetic structure of the Danish wild boars. Wild boars from Klelund Plantation do not show any apparent fitness costs from an extremely small founder base and living in isolation. However, there may be hidden costs related to their high level of inbreeding and having the longest ROHs found in pigs and wild boars globally. Such hidden costs of inbreeding may only become apparent when these inbred animals have to compete directly with less inbred conspecifics. With the use of genomic analysis, those hidden costs can be unravelled, for instance by inferring if deleterious high‐frequency alleles are present. In the current situation, the Klelund population seems to thrive. However, with changing environments low diversity may result in lower potential to adapt. All in all, the Klelund population has strong potential to untangle how small populations with high levels of inbreeding succeed to persist.

## CONFLICT OF INTEREST

The authors have no conflict of interest to declare.

## Supporting information

Supplementary Material

Table S4

## Data Availability

The data for this study have been deposited in the Dryad Digital Repository: https://doi.org/10.5061/dryad.905qfttnc.

## References

[eva13385-bib-0001] Aguilar, A. , Roemer, G. , Debenham, S. , Binns, M. , Garcelon, D. , & Wayne, R. K. (2004). High MHC diversity maintained by balancing selection in an otherwise genetically monomorphic mammal. Proceedings of the National Academy of Sciences of the United States of America, 101(10), 3490–3494. 10.1073/pnas.0306582101 14990802 PMC373489

[eva13385-bib-0002] Alban, L. , Andersen, M. M. , Asferg, T. , Boklund, A. , Fernandez, N. , Goldbach, S. G. , Greiner, M. , Hojgaard, A. , Kramer‐Schadt, S. , Stockmarr, A. , Thulke, H. H. , Uttenthal, Å. , & Ydesen, B. (2005). Classical swine fever and wild boar in Denmark : a risk analysis.

[eva13385-bib-0003] Alexander, D. H. , & Lange, K. (2011). Enhancements to the ADMIXTURE algorithm for individual ancestry estimation. BMC Bioinformatics, 12. 10.1186/1471-2105-12-246 PMC3146885 21682921

[eva13385-bib-0004] Alexandri, P. , Megens, H.‐J. , Crooijmans, R. P. M. A. , Groenen, M. A. M. , Goedbloed, D. J. , Herrero‐Medrano, J. M. , Rund, L. A. , Schook, L. B. , Chatzinikos, E. , Triantaphyllidis, C. , & Triantafyllidis, A. (2017). Distinguishing migration events of different timing fo r wild boar in the balkans. Journal of Biogeography, 44(2), 259–270. 10.1111/jbi.12861

[eva13385-bib-0005] Alves, C. , Paulo, I. P. , Godinho, R. , Vicente, J. , Gortázar, C. , & Scandura, M. (2010). Genetic diversity of wild boar populations and domestic pig breeds (Sus Scrofa) in South‐Western Europe. Biological Journal of the Linnean Society, 101(4), 797–822. 10.1111/j.1095-8312.2010.01530.x

[eva13385-bib-0006] Apollonio, M. , Randi, E. , & Toso, S. (1988). The systematics of the wild boar (Sus Scrofa l.) in Italy. Bolletino Di Zoologia, 55(1–4), 213–221. 10.1080/11250008809386619

[eva13385-bib-0007] Auton, A. , Bryc, K. , Boyko, A. R. , Lohmueller, K. E. , Novembre, J. , Reynolds, A. , Indap, A. , Wright, M. H. , Degenhardt, J. D. , Gutenkunst, R. N. , King, K. S. , Nelson, M. R. , & Bustamante, C. D. (2009). Global distribution of genomic diversity underscores rich complex history of continental human populations. Genome Research, 19(5), 795–803. 10.1101/gr.088898.108 19218534 PMC2675968

[eva13385-bib-0008] Barrett, S. C. H. , & Charlesworth, D. (1991). Effects of a change in the level of inbreeding on the genetic load. Nature, 352(6335), 522–524. 10.1038/352522a0 1865906

[eva13385-bib-0009] Boakes, E. , & Wang, J. (2005). A simulation study on detecting purging of inbreeding depression in captive populations. Genetical Research, 86(2), 139–148. 10.1017/S001667230500772X 16356287

[eva13385-bib-0010] Bortoluzzi, C. , Bosse, M. , Derks, M. F. L. , Crooijmans, R. P. M. A. , Groenen, M. A. M. , & Megens, H.‐J. (2020). The type of bottleneck matters: Insights into the deleterious variation landscape of small managed populations. Evolutionary Applications, 13(2), 330–341. 10.1111/eva.12872 31993080 PMC6976952

[eva13385-bib-0011] Bosse, M. , Madsen, O. , Megens, H.‐J. , Frantz, L. A. F. , Paudel, Y. , Crooijmans, R. P. M. A. , & Groenen, M. A. M. (2015). Hybrid origin of European commercial pigs examined by an in‐depth haplotype analysis on chromosome 1. Frontiers in Genetics, 5, 1–9. 10.3389/fgene.2014.00442 PMC4283659 25601878

[eva13385-bib-0012] Bosse, M. , Megens, H. J. , Derks, M. F. L. , de Cara, Á. M. R. , & Groenen, M. A. M. (2019). Deleterious alleles in the context of domestication, inbreeding, and selection. Evolutionary Applications, 12(1), 6–17. 10.1111/eva.12691 30622631 PMC6304688

[eva13385-bib-0013] Bosse, M. , Megens, H. J. , Madsen, O. , Paudel, Y. , Frantz, L. A. F. , Schook, L. B. , Crooijmans, R. P. M. A. , & Groenen, M. A. M. (2012). Regions of homozygosity in the porcine genome: Consequence of demography and the recombination landscape. PLoS Genetics, 8(11), 10.1371/journal.pgen.1003100 PMC3510040 23209444

[eva13385-bib-0014] Chang, C. C. , Chow, C. C. , Tellier, L. C. A. M. , Vattikuti, S. , Purcell, S. M. , & Lee, J. J. (2015). Second‐generation PLINK: Rising to the challenge of larger and richer datasets. GigaScience, 4(1), 1–16. 10.1186/s13742-015-0047-8 25722852 PMC4342193

[eva13385-bib-0015] Crnokrak, P. , & Roff, D. A. (1999). Inbreeding depression in the wild. Heredity, 83(3), 260–270. 10.1038/sj.hdy.6885530 10504423

[eva13385-bib-0016] Curik, I. , Ferenčaković, M. , & Sölkner, J. (2014). Inbreeding and runs of homozygosity: A possible solution to an old problem. Livestock Science, 166, 26–34. 10.1016/j.livsci.2014.05.034

[eva13385-bib-0017] Dadi, H. , Le, M. T. , Dinka, H. , Nguyen, D. T. , Choi, H. , Cho, H. , Choi, M. , Kim, J. H. , Park, J. K. , Soundrarajan, N. , & Park, C. (2015). Genetic diversity and MRNA expression of porcine MHC class i chain‐related 2 (SLA‐MIC2) gene and development of a high‐resolution typing method. PLoS One, 10(8), 1–17. 10.1371/journal.pone.0135922 PMC4549063 26305091

[eva13385-bib-0018] de Jong, J. F. (2018). Genetic variation of wildlife in a human‐dominated landscape: Genome‐wide SNP analysis of wild boar (Sus Scrofa) and red deer (Cervus Elaphus) from the European continent. Wageningen University & Research.

[eva13385-bib-0019] Ferenčaković, M. , Hamzić, E. , Gredler, B. , Solberg, T. R. , Klemetsdal, G. , Curik, I. , & Sölkner, J. (2013). Estimates of autozygosity derived from runs of homozygosity: Empirical evidence from selected cattle populations. Journal of Animal Breeding and Genetics, 130(4), 286–293. 10.1111/jbg.12012 23855630

[eva13385-bib-0020] Frankham, R. (2010). Challenges and opportunities of genetic approaches to biological conservation. Biological Conservation, 143(9), 1919–1927. 10.1016/j.biocon.2010.05.011

[eva13385-bib-0021] Frankham, R. , Lees, K. , Montgomery, M. E. , England, P. R. , Lowe, E. H. , & Briscoe, D. A. (1999). Do population size bottlenecks reduce evolutionary potential? Animal Conservation, 2(4), 255–260. 10.1111/j.1469-1795.1999.tb00071.x

[eva13385-bib-0022] Frantz, A. C. , Zachos, F. E. , Kirschning, J. , Cellina, S. , Bertouille, S. , Mamuris, Z. , Koutsogiannouli, E. A. , & Burke, T. (2013). Genetic evidence for introgression between domestic pigs and wild boars (Sus Scrofa) in Belgium and Luxembourg: A comparative approach with multiple marker systems: Introgression between pigs and boars. Biological Journal of the Linnean Society, 110(1), 104–115. 10.1111/bij.12111

[eva13385-bib-0023] Frauendorf, M. , Gethöffer, F. , Siebert, U. , & Keuling, O. (2016). The influence of environmental and physiological factors on the litter size of wild boar (Sus Scrofa) in an agriculture dominated area in Germany. Science of the Total Environment, 541, 877–882. 10.1016/j.scitotenv.2015.09.128 26437356

[eva13385-bib-0024] Gethöffer, F. , Sodeikat, G. , & Pohlmeyer, K. (2007). Reproductive parameters of wild boar (Sus Scrofa) in three different parts of Germany. European Journal of Wildlife Research, 53(4), 287–297. 10.1007/s10344-007-0097-z

[eva13385-bib-0025] Goedbloed, D. J. , van Hooft, P. , Megens, H.‐J. , Langenbeck, K. , Lutz, W. , Crooijmans, R. P. M. A. , van Wieren, S. E. , Ydenberg, R. C. , & Prins, H. H. T. (2013). Reintroductions and genetic introgression from domestic pigs have shaped the genetic population structure of northwest European wild boar. BMC Genetics, 14(1), 43. 10.1186/1471-2156-14-43 23688182 PMC3663677

[eva13385-bib-0026] Goodnight, C. J. (1988). Epistasis and the effect of founder events on the additive genetic variance. Evolution, 42(3), 441–454. 10.1111/j.1558-5646.1988.tb04151.x 28564006

[eva13385-bib-0027] Groenen, M. A. M. , Archibald, A. L. , Uenishi, H. , Tuggle, C. K. , Takeuchi, Y. , Rothschild, M. F. , Rogel‐Gaillard, C. , Park, C. , Milan, D. , Megens, H. J. , Li, S. , Larkin, D. M. , Kim, H. , Frantz, L. A. F. , Caccamo, M. , Ahn, H. , Aken, B. L. , Anselmo, A. , Anthon, C. , … Schook, L. B. (2012). Analyses of pig genomes provide insight into porcine demography and evolution. Nature, 491(7424), 393–398. 10.1038/nature11622 23151582 PMC3566564

[eva13385-bib-0028] Grossen, C. , Guillaume, F. , Keller, L. F. , & Croll, D. (2020). Purging of highly deleterious mutations through severe bottlenecks in alpine ibex. Nature Communications, 11(1), 1001. 10.1038/s41467-020-14803-1 PMC7035315 32081890

[eva13385-bib-0029] Hald‐Mortensen, P. , Gregersen, J. , Skriver, J. , & Naturfond, AvJ. (2012). Tofte Skov Og Mose: Status 2012. U.S. Government Printing Office.

[eva13385-bib-0030] Hedrick, P. W. , & Kalinowski, S. T. (2000). Inbreeding depression in conservation biology. Annual Review of Ecology and Systematics, 31(1), 139–162. 10.1146/annurev.ecolsys.31.1.139

[eva13385-bib-0031] Herrero‐Medrano, J. M. , Megens, H. J. , Groenen, M. A. M. , Ramis, G. , Bosse, M. , Pérez‐Enciso, M. , & Crooijmans, R. P. M. A. (2013). Conservation genomic analysis of domestic and wild pig populations from the Iberian Peninsula. BMC Genetics, 14, 1–13. 10.1186/1471-2156-14-106 24172017 PMC3840735

[eva13385-bib-0032] Humble, E. , Dobrynin, P. , Senn, H. , Chuven, J. , Scott, A. F. , Mohr, D. W. , Dudchenko, O. , Omer, A. D. , Colaric, Z. , Aiden, E. L. , Dhaheri, S. S. A. , Wildt, D. , Oliaji, S. , Tamazian, G. , Pukazhenthi, B. , Ogden, R. , & Koepfli, K.‐P. (2020). Chromosomal‐level genome assembly of the scimitar‐horned oryx: Insights into diversity and demography of a species extinct in the wild. Molecular Ecology Resources, 20(6), 1668–1681. 10.1111/1755-0998.13181 32365406 PMC10332132

[eva13385-bib-0033] Iacolina, L. , Pertoldi, C. , Amills, M. , Kusza, S. , Megens, H.‐J. , Bâlteanu, V. A. , Bakan, J. , Cubric‐Curik, V. , Oja, R. , Saarma, U. , Scandura, M. , Šprem, N. , & Stronen, A. V. (2018). Hotspots of recent hybridization between pigs and wild boars in Europe. Scientific Reports, 8(1). 10.1038/s41598-018-35865-8 PMC6255867 30478374

[eva13385-bib-0034] Iacolina, L. , Scandura, M. , Goedbloed, D. J. , Alexandri, P. , Crooijmans, R. P. M. A. , Larson, G. , Archibald, A. , Apollonio, M. , Schook, L. B. , Groenen, M. A. M. , & Megens, H. J. (2016). Genomic diversity and differentiation of a managed island wild boar population. Heredity, 116(1), 60–67. 10.1038/hdy.2015.70 26243137 PMC4675874

[eva13385-bib-0035] Keller, L. F. , & Waller, D. M. (2002). Inbreeding effects in wild populations. Trends in Ecology and Evolution, 17(5), 230–241. 10.1016/S0169-5347(02)02489-8

[eva13385-bib-0036] Keller, M. C. , Visscher, P. M. , & Goddard, M. E. (2011). Quantification of inbreeding due to distant ancestors and its detection using dense single nucleotide polymorphism data. Genetics, 189(1), 237–249. 10.1534/genetics.111.130922 21705750 PMC3176119

[eva13385-bib-0037] Kim, E. S. , Sonstegard, T. S. , Van Tassell, C. P. , Wiggans, G. , & Rothschild, M. F. (2015). The relationship between runs of homozygosity and inbreeding in jersey cattle under selection. PLoS One, 10(7), 1–17. 10.1371/journal.pone.0129967 PMC4496098 26154171

[eva13385-bib-0038] Kirin, M. , McQuillan, R. , Franklin, C. S. , Campbell, H. , Mckeigue, P. M. , & Wilson, J. F. (2010). Genomic runs of homozygosity record population history and consanguinity. PLoS One, 5(11), 1–7. 10.1371/journal.pone.0013996 PMC2981575 21085596

[eva13385-bib-0039] Kyriazis, C. , Wayne, R. K. , & Lohmueller, K. E. (2019). High genetic diversity can contribute to extinction in small populations. BioRxiv, 12.

[eva13385-bib-0040] Kyriazis, C. C. , Wayne, R. K. , & Lohmueller, K. E. (2020). Strongly deleterious mutations are a primary determinant of extinction risk due to inbreeding depression. Evolution Letters, 5(1), 33–47. 10.1002/evl3.209 33552534 PMC7857301

[eva13385-bib-0041] Lacy, R. C. (2000). Considering threats to the viability of small populations using individual‐based models. Ecological Bulletins, 48, 39–51.

[eva13385-bib-0042] Latch, E. K. (2020). Integrating genomics into conservation management. Molecular Ecology Resources, 20(6), 1455–1457. 10.1111/1755-0998.13188 32416033

[eva13385-bib-0043] Lawson, D. J. , van Dorp, L. , & Falush, D. (2018). A Tutorial on how not to over‐interpret STRUCTURE and ADMIXTURE bar plots. Nature Communications, 9(1), 1–11. 10.1038/s41467-018-05257-7 PMC6092366 30108219

[eva13385-bib-0044] Leppälä, K. , Nielsen, S. V. , & Mailund, T. (2017). Admixturegraph: An R package for admixture graph manipulation and fitting. Bioinformatics, 33(11), 1738–1740. 10.1093/bioinformatics/btx048 28158333 PMC5447235

[eva13385-bib-0045] Lindqvist, C. , & Rajora, O. P. (2019). Paleogenomics: Genome‐scale analysis of ancient DNA. In C. Lindqvist , O. P. Rajora .

[eva13385-bib-0046] Liu, L. , Bosse, M. , Megens, H.‐J. , Frantz, L. A. F. , Lee, Y.‐L. , Irving‐Pease, E. K. , Narayan, G. , Groenen, M. A. M. , & Madsen, O. (2019). Genomic analysis on Pygmy hog reveals extensive interbreeding during wild boar expansion. Nature Communications, 10(1), 1992. 10.1038/s41467-019-10017-2 PMC6491599 31040280

[eva13385-bib-0047] Ma, J. , & Amos, C. I. (2012). Principal components analysis of population admixture. PLoS One, 7(7). 10.1371/journal.pone.0040115 PMC3392282 22808102

[eva13385-bib-0048] Malmsten, A. , Jansson, G. , Lundeheim, N. , & Dalin, A.‐M. (2017). The reproductive pattern and potential of free ranging female wild boars (Sus Scrofa) in Sweden. Acta Veterinaria Scandinavica, 59(1), 52. 10.1186/s13028-017-0321-0 28764737 PMC5539618

[eva13385-bib-0049] Mastrangelo, S. , Tolone, M. , Sardina, M. T. , Sottile, G. , Sutera, A. M. , Di Gerlando, R. , & Portolano, B. (2017). Genome‐wide scan for runs of homozygosity identifies potential candidate genes associated with local adaptation in Valle Del Belice Sheep. Genetics Selection Evolution, 49(1). 10.1186/s12711-017-0360-z PMC5684758 29137622

[eva13385-bib-0050] Mathur, S. , Tomeček, J. , Tarango‐Arámbula, L. , Perez, R. , & DeWoody, A. (2021). An evolutionary perspective on contemporary genetic load in threatened species to inform future conservation efforts. Preprints. 10.22541/au.162495929.94655412/v1

[eva13385-bib-0051] Meyermans, R. , Gorssen, W. , Buys, N. , & Janssens, S. (2020). How to study runs of homozygosity using PLINK? A guide for analyzing medium density SNP data in livestock and pet species. BMC Genomics, 21(1), 94. 10.1186/s12864-020-6463-x 31996125 PMC6990544

[eva13385-bib-0052] Muzzio, M. , Motti, J. M. B. , Paz Sepulveda, P. B. , Yee, M.‐C. , Cooke, T. , Santos, M. R. , Ramallo, V. , Alfaro, E. L. , Dipierri, J. E. , Bailliet, G. , Bravi, C. M. , Bustamante, C. D. , & Kenny, E. E. (2018). Population structure in Argentina. PLoS One, 13(5), 1–13. 10.1371/journal.pone.0196325 PMC5929549 29715266

[eva13385-bib-0053] Novembre, J. , Johnson, T. , Bryc, K. , Kutalik, Z. , Boyko, A. R. , Auton, A. , Indap, A. , King, K. S. , Bergmann, S. , Nelson, M. R. , Stephens, M. , & Bustamante, C. D. (2008). Genes mirror geography within Europe. Nature, 456(7218), 98–101. 10.1038/nature07331 18758442 PMC2735096

[eva13385-bib-0054] Oliver, M. K. , Telfer, S. , & Piertney, S. B. (2009). Major histocompatibility complex (MHC) heterozygote superiority to natural multi‐parasite infections in the Water Vole (Arvicola Terrestris). Proceedings of the Royal Society B: Biological Sciences, 276(1659), 1119–1128. 10.1098/rspb.2008.1525 PMC2679068 19129114

[eva13385-bib-0055] Orłowska, L. , Rembacz, W. , & Florek, C. (2013). Carcass weight, condition and reproduction of wild boar harvested in North‐Western Poland: Carcass weight, condition and reproduction of wild boar in Poland. Pest Management Science, 69(3), 367–370. 10.1002/ps.3355 22848027

[eva13385-bib-0056] Parisod, C. , Trippi, C. , & Galland, N. (2005). Genetic variability and founder effect in the pitcher plant Sarracenia Purpurea (Sarraceniaceae) in populations introduced into Switzerland: From inbreeding to invasion. Annals of Botany, 95(2), 277–286. 10.1093/aob/mci023 15546932 PMC4246826

[eva13385-bib-0057] Pecnerova, P. (2018). Genomic analysis of the process leading up to the extinction of the Woolly Mammoth. Stockholm Univeristy.

[eva13385-bib-0058] Purfield, D. C. , McParland, S. , Wall, E. , & Berry, D. P. (2017). The distribution of runs of homozygosity and selection signatures in six commercial meat sheep breeds. PLoS One, 12(5), 1–23. 10.1371/journal.pone.0176780 PMC5413029 28463982

[eva13385-bib-0059] R Core Team . (2021). R: A language and environment for statistical computing. R Foundation for Statistical Computing.

[eva13385-bib-0060] Ramos, A. M. , Crooijmans, R. P. M. A. , Affara, N. A. , Amaral, A. J. , Archibald, A. L. , Beever, J. E. , Bendixen, C. , Churcher, C. , Clark, R. , Dehais, P. , Hansen, M.S. , Hedegaard, J. , Hu, Z.‐L. , Kerstens, H. H. , Law, A. S. , Megens, H.‐J. , Milan, D. , Nonneman, D. J. , Rohrer, G. A. , … Groenen, M. A. M. (2009). Design of a high density SNP genotyping assay in the pig using SNPs identified and characterized by next generation sequencing technology. PLoS One, 4(8), e6524. 10.1371/journal.pone.0006524 19654876 PMC2716536

[eva13385-bib-0061] Robinson, J. A. , Brown, C. , Kim, B. Y. , Lohmueller, K. E. , & Wayne, R. K. (2018). Purging of strongly deleterious mutations explains long‐term persistence and absence of inbreeding depression in island foxes. Current Biology, 28(21), 3487–3494.e4. 10.1016/j.cub.2018.08.066 30415705 PMC6462144

[eva13385-bib-0062] Robinson, J. A. , Räikkönen, J. , Vucetich, L. M. , Vucetich, J. A. , Peterson, R. O. , Lohmueller, K. E. , & Wayne, R. K. (2019). Genomic signatures of extensive inbreeding in Isle Royale Wolves, a population on the threshold of extinction. Science Advances, 5. 10.1126/sciadv.aau0757 PMC6541468 31149628

[eva13385-bib-0063] Saccheri, I. , Kuussaari, M. , Kankare, M. , Vikman, P. , Fortelius, W. , & Hanski, I. (1998). Inbreeding and extinction in a butterfly metapopulation. Nature, 392(6675), 491–494. 10.1038/33136

[eva13385-bib-0064] Santos, P. S. C. , Courtiol, A. , Heidel, A. J. , Höner, O. P. , Heckmann, I. , Nagy, M. , Mayer, F. , Platzer, M. , Voigt, C. C. , & Sommer, S. (2016). MHC‐dependent mate choice is linked to a trace‐amine‐associated receptor gene in a mammal. Scientific Reports, 6(1), 38490. 10.1038/srep38490 27941813 PMC5150237

[eva13385-bib-0065] Scandura, M. , Iacolina, L. , & Apollonio, M. (2011). Genetic diversity in the European wild boar Sus Scrofa: Phylogeography, population structure and wild x domestic hybridization. Mammal Review, 41(2), 125–137. 10.1111/j.1365-2907.2010.00182.x

[eva13385-bib-0066] Scandura, M. , Iacolina, L. , Crestanello, B. , Pecchioli, E. , Di Benedetto, M. F. , Russo, V. , Davoli, R. , Apollonio, M. , & Bertorelle, G. (2008). Ancient vs. recent processes as factors shaping the genetic variation of the European wild boar: Are the effects of the last glaciation still detectable? Molecular Ecology, 17(7), 1745–1762. 10.1111/j.1365-294X.2008.03703.x 18371016

[eva13385-bib-0067] Schwartz, J. C. , Hemmink, J. D. , Graham, S. P. , Tchilian, E. , Charleston, B. , Hammer, S. E. , Ho, C. S. , & Hammond, J. A. (2018). The major histocompatibility complex homozygous inbred babraham pig as a resource for veterinary and translational medicine. HLA, 92(1), 40–43. 10.1111/tan.13281 PMC6099331 29687612

[eva13385-bib-0068] Simons, Y. B. , & Sella, G. (2016). The impact of recent population history on the deleterious mutation load in humans and close evolutionary relatives. Current Opinion in Genetics and Development, 41, 150–158. 10.1016/j.physbeh.2017.03.040 27744216 PMC5161708

[eva13385-bib-0069] Skotte, L. , Korneliussen, T. S. , & Albrechtsen, A. (2013). Estimating individual admixture proportions from next generation sequencing data. Genetics, 195(3), 693–702. 10.1534/genetics.113.154138 24026093 PMC3813857

[eva13385-bib-0070] van der Valk, T. , de Manuel, M. , Marquès‐Bonet, T. , & Guschanski, K. (2019). Estimates of genetic load in small populations suggest extensive purging of deleterious alleles. BioRxiv. 10.1101/696831

[eva13385-bib-0071] Vernesi, C. , Crestanello, B. , Pecchioli, E. , Tartari, D. , Caramelli, D. , Hauffe, H. , & Bertorelle, G. (2003). The genetic impact of demographic decline and reintroduction in the wild boar (Sus Scrofa): A microsatellite analysis. Molecular Ecology, 12(3), 585–595. 10.1046/j.1365-294X.2003.01763.x 12675815

[eva13385-bib-0072] Vilaça, S. T. , Biosa, D. , Zachos, F. , Iacolina, L. , Kirschning, J. , Alves, P. C. , Paule, L. , Gortazar, C. , Mamuris, Z. , Jędrzejewska, B. , Borowik, T. , Sidorovich, V. E. , Kusak, J. , Costa, S. , Schley, L. , Hartl, G. B. , Apollonio, M. , Bertorelle, G. , & Scandura, M. (2014). “Mitochondrial phylogeography of the European wild boar: The effect of climate on genetic diversity and spatial lineage sorting across Europe” edited by P. Linder. Journal of Biogeography, 41(5), 987–998. 10.1111/jbi.12268

[eva13385-bib-0073] Wahl, L. M. , Gerrish, P. J. , & Saika‐Voivod, I. (2002). Evaluating the impact of population bottlenecks in experimental evolution. Genetics, 162(2), 961–971. 10.1093/genetics/162.2.961 12399403 PMC1462272

[eva13385-bib-0074] Wang, P. , Burley, J. T. , Liu, Y. , Chang, J. , Chen, D. E. , Qi, L. U. , Li, S.‐H. , Zhou, X. , Edwards, S. , & Zhang, Z. (2021). “Genomic consequences of long‐term population decline in brown eared pheasant” edited by F. Wei. Molecular Biology and Evolution, 38(1), 263–273. 10.1093/molbev/msaa213 32853368 PMC7783171

[eva13385-bib-0075] Wilson, B. A. , Petrov, D. A. , & Messer, P. W. (2014). Soft selective sweeps in complex demographic scenarios. Genetics, 198(2), 669–684. 10.1534/genetics.114.165571 25060100 PMC4266194

[eva13385-bib-0076] Xue, Y. , Prado‐Martinez, J. , Sudmant, P. H. , Narasimhan, V. , Ayub, Q. , Szpak, M. , Frandsen, P. , Chen, Y. , Yngvadottir, B. , Cooper, D. N. , De Manuel, M. , Hernandez‐Rodriguez, J. , Lobon, I. , Siegismund, H. R. , Pagani, L. , Quail, M. A. , Hvilsom, C. , Mudakikwa, A. , Eichler, E. E. , … Scally, A. (2015). Mountain gorilla genomes reveal the impact of long‐term population decline and inbreeding. Science, 348(6231), 242–245. 10.1126/science.aaa3952 25859046 PMC4668944

[eva13385-bib-0077] Yang, B. , Cui, L. , Perez‐Enciso, M. , Traspov, A. , Crooijmans, R. P. M. A. , Zinovieva, N. , Schook, L. B. , Archibald, A. , Gatphayak, K. , Knorr, C. , Triantafyllidis, A. , Alexandri, P. , Semiadi, G. , Hanotte, O. , Dias, D. , Dovč, P. , Uimari, P. , Iacolina, L. , Scandura, M. , … Megens, H. J. (2017a). Genome‐wide SNP data unveils the globalization of domesticated pigs. Genetics Selection Evolution, 49(1), 1–15. 10.1186/s12711-017-0345-y PMC5609043 28934946

[eva13385-bib-0078] Yang, B. , Cui, L. , Perez‐Enciso, M. , Traspov, A. , Crooijmans, R. P. M. A. , Zinovieva, N. , Schook, L. B. , Archibald, A. , Gatphayak, K. , Knorr, C. , Triantafyllidis, A. , Alexandri, P. , Semiadi, G. , Hanotte, O. , Dias, D. , Dovč, P. , Uimari, P. , Iacolina, L. , Scandura, M. , … Megens, H. J. (2017b). Genome‐wide SNP data unveils the globalization of domesticated pigs. Genetics Selection Evolution, 49(1), 1–15. 10.1186/s12711-017-0345-y PMC5609043 28934946

[eva13385-bib-0079] Zhang, Q. , Guldbrandtsen, B. , Bosse, M. , Lund, M. S. , & Sahana, G. (2015). Runs of homozygosity and distribution of functional variants in the cattle genome. BMC Genomics, 16(1), 1–16. 10.1186/s12864-015-1715-x 26198692 PMC4508970

[eva13385-bib-0080] Zheng, X. , Levine, D. , Shen, J. , Gogarten, S. M. , Laurie, C. , & Weir, B. S. (2012). A high‐performance computing toolset for relatedness and principal component analysis of SNP data. Bioinformatics, 28(24), 3326–3328. 10.1093/bioinformatics/bts606 23060615 PMC3519454

