# Supplementary material for: Genomic consequences of a century of inbreeding and isolation in the Danish wild boar population
Source: Evol Appl. 2022 May 17;15(6):954–66. doi: 10.1111/eva.13385 (PMC9234630; doi:10.1111/eva.13385)
Supplement: Supplementary file 1 — Supplementary Material [file EVA-15-954-s002.doc]

Supplemental Information for:

**Genomic consequences of a century of inbreeding and isolation in the Danish wild boar population**

Beril Yıldız^1,2^, Hendrik-Jan Megens^1^, Christina Hvilsom^3^*, Mirte Bosse^1,4^*

**Table of Contents:**

| **Supplementary Figure 1** | Page 2 |
| --- | --- |
| **Supplementary Figure 2** | Page 3 |
| **Supplementary Figure 3** | Page 4 |
| **Supplementary Figure 4** | Page 5 |
| **Supplementary Figure 5** | Page 6 |
| **Supplementary Table 1** | Page 6 |
| **Supplementary Table 2** | Page 7 |
| **Supplementary Table 3** | Page 8 |

**Suppl. Figure 1.** Chromosomal distribution of the ROH segments of the study population consisting of wild boars from Ulm and Klelund Plantation, Denmark. The x-axis is scaled according to the length of each chromosome. Individuals from the Danish population are highlighted as red, whereas individuals from Ulm are highlighted as blue.

**Suppl. Figure 2.** Principal component analysis (PCA) of 459 wild boars across Europe including the study populations Klelund, Denmark and Ulm, Germany using 43,121 SNPS. The fraction of the total variance explained was 51.03% for eigenvector 1 (PC1) and 12.4% for eigenvector 2 (PC2).

**Suppl. Figure 3.** The percent ROH frequency and total ROH length (Mb) of the global pig data under different ROH length categories.

**Suppl. Figure 4.** Admixture plot of K=2 to K=8 showing the ancestry proportions of 459 individuals from European wild boar populations.

**Suppl. Figure 5.** Cross validation error over different K-values of the European admixture plot.

**Suppl. Table 1. Pairwise estimation of F_ST_ between clusters based on the inferred allele frequencies in the global pig data and the study populations (N=1263) suggested by Admixture.**

|  | CLUSTER**1** | CLUSTER**2** | CLUSTER**3** |
| --- | --- | --- | --- |
| Cluster**1** |  |  |  |
| CLUSTER**2** | 0.431 |  |  |
| CLUSTER**3** | 0.312 | 0.169 |  |
| CLUSTER**4** | 0.354 | 0.170 | 0.114 |

**Suppl. Table 2.** ROH characteristics for each individual in the study populations. The results of the ROH analysis of 11 individuals from Ulm, Germany and 21 individuals from Klelund Plantation, Denmark including total ROH number, total ROH length, average ROH length, and genomic inbreeding coefficient (FROH) per individual. ROH lengths are shown in Mb.

| Population | ROH number | Total ROH length | Average ROH length | F_ROH_ |
| --- | --- | --- | --- | --- |
|  | 16 | 111.6 | 6.97 | 0.05 |
|  | 11 | 116.8 | 10.62 | 0.05 |
|  | 19 | 163.8 | 8.62 | 0.07 |
|  | 18 | 174.3 | 9.68 | 0.07 |
|  | 18 | 219.6 | 12.20 | 0.09 |
| Ulm, | 23 | 246.1 | 10.70 | 0.10 |
| Germany | 21 | 258.0 | 12.29 | 0.11 |
|  | 23 | 328.6 | 14.29 | 0.13 |
|  | 28 | 401.2 | 14.33 | 0.16 |
|  | 30 | 431.3 | 14.38 | 0.18 |
|  | 30 | 674.4 | 22.48 | 0.28 |
|  | 112 | 1048.8 | 9.36 | 0.43 |
|  | 81 | 1215.0 | 15.00 | 0.50 |
|  | 82 | 1239.1 | 15.11 | 0.51 |
|  | 113 | 1244.4 | 11.01 | 0.51 |
|  | 86 | 1254.8 | 14.59 | 0.52 |
|  | 91 | 1278.4 | 14.05 | 0.52 |
|  | 115 | 1293.0 | 11.24 | 0.53 |
|  | 80 | 1344.2 | 16.80 | 0.55 |
|  | 97 | 1354.3 | 13.96 | 0.56 |
| Klelund | 90 | 1381.3 | 15.35 | 0.57 |
| Plantation, | 84 | 1384.2 | 16.48 | 0.57 |
| Denmark | 83 | 1391.8 | 16.77 | 0.57 |
|  | 81 | 1395.7 | 17.23 | 0.57 |
|  | 93 | 1445.9 | 15.55 | 0.59 |
|  | 92 | 1459.5 | 15.86 | 0.60 |
|  | 92 | 1483.7 | 16.13 | 0.61 |
|  | 88 | 1512.2 | 17.18 | 0.62 |
|  | 84 | 1533.6 | 18.26 | 0.63 |
|  | 83 | 1546.4 | 18.63 | 0.63 |
|  | 82 | 1615.2 | 19.70 | 0.66 |
|  | 91 | 1633.6 | 17.95 | 0.67 |

**Suppl. Table 3.** Phenotypic traits of the wild boars in Klelund, Denmark and other European wild boar populations. For each European population, the average litter size, percentage of piglet survival, and the source is shown. Missing data are shown with asterisk. Modified from Sam X, MSc thesis report.

| **Population** | **Litter size** | **Piglet survival** | **Source** |
| --- | --- | --- | --- |
| **Denmark Klelund** | 4.5 | 81.8 % | Questionnaire |
| **Denmark Tofte** | 5.5 | 36.4% | Questionnaire |
| **France East** | 5.2 | 87% | (Celina, 2008) |
| **France West** | 4.6 | 86% | (Celina, 2008) |
| **Germany Central** | 4.7 | 91.6% | (Cellina, 2008) |
| **Germany North-East** | 5.5 | 84% | (Cellina, 2008) |
| **Germany North-West** | 6.9 | 88% | (Fernández-Llario & Mateos-Quesada, 1998; Frauendorf et al., 2016) |
| **Germany South-West** | 5.6 | 84% | (Fernández-Llario & Mateos-Quesada, 1998) |
| **Germany West** | 5.3 | 84% | (Fernández-Llario & Mateos-Quesada, 1998) |
| **Hungary** | 6.7 | 83% | (Náhlik & Sándor, 2003) |
| **Italy Central** | 4.9 | * | (Celina, 2008) |
| **Italy North** | 4.7 | * | (Celina, 2008) |
| **Italy North-West** | 4.9 | * | (Celina, 2008) |
| **Italy South** | 3.7 | * | (Celina, 2008) |
| **Luxembourg** | 5.3 | 82% | (Celina, 2008) |
| **Netherlands** | 4.5 | * (>65%) | (Groot Bruinderink et al., 1994) and Questionnaire |
| **Poland** | 5.6 | * | (Fernández-Llario & Mateos-Quesada, 1998; Orłowska et al., 2012) |
| **Poland West** | 4.8 | * | (Celina, 2008) |
| **Portugal** | 4.1 | 93.7% | (Fonseca et al., 2004, 2010) |
| **Spain South-East** | 4.1 | 69.4% | (Celina, 2008) |
| **Spain Western** | 3.5 | * | (Fernández-Llario & Mateos-Quesada, 1998; Fernández-Llario et al., 2004; Malmsten et al., 2017) |
| **Sweden** | 5.4 | * | (Malmsten et al., 2017; Malmsten & Dalin, 2015) |
| **Switzerland** | 4.8 | * | (Malmsten et al., 2017) |
